# Explorations on people centredness in health systems

**DOI:** 10.1093/heapol/czu082

**Published:** 2014-09-11

**Authors:** Kabir Sheikh, Michael Kent Ranson, Lucy Gilson

**Affiliations:** ^1^Public Health Foundation of India, New Delhi, India, ^2^Alliance for Health Policy and Systems Research, World Health Organization, Geneva, Switzerland, ^3^Health Policy and Systems Division, School of Public Health and Family Medicine, University of Cape Town, Cape Town, South Africa and ^4^Health Economics and Systems Analysis Group, Department of Global Health and Development, London School of Hygiene and Tropical Medicine, London, UK

## Introduction

Health systems should ultimately seek to serve people and society. They must aim to bring value in people’s lives not only by caring for them when sick or giving support to prevent or limit illness and its effects, but also, more broadly, by offering the promise of economic security to all for times of great vulnerability.

Health systems are also human systems. At their heart is a personal encounter, the interaction between the patient and the health provider—sometimes tenuous, often contested, but always with the potential for humanity and compassion. But many different types of people—individuals, groups and communities—make up health systems, ‘live’ within them, have roles, stakes and power in them, and are central to their existence and functioning. People make all the most important decisions in health systems—either by accessing services as patients, setting rules and allocating resources as policymakers, or enacting, coping with and subverting those rules, as implementers, managers, providers and service users. Communities and citizens influence these systems by shaping the social norms and contexts in which they operate. Community norms and behaviour drive health market forces and practices, influence how individuals and families access services, and can help hold systems accountable. Citizens may also influence system development through their electoral voting power, exercising the ‘long route’ to accountability.

People centredness embraces this essentially human character of health systems. Yet, the term is surprisingly new in health system debate and the common response to its use is ‘what does that mean?’ This supplement advances the conversation by exploring varied perspectives on the concept of people centred health systems (PCHS). PCHS emerges as a multi-faceted concept, with ideological power and also carrying huge potential for practical thinking and change in health systems. While Universal Health Coverage has become emblematic globally for health systems change for better health care access and quality, and social protection, PCHS offers opportunities to elaborate and deepen our understanding of what such change should entail in the operational practices of health systems.

The initial 11 articles in this collection, published as a printed supplement, begin to illustrate different aspects of the PCHS concept (further articles on the theme will be released in an online collection, and will be scattered through subsequent print editions of the journal). Four overarching themes that define and represent different aspects of PCHS emerge from this set of articles, and from other existing writing on PCHS and related themes. These aspects are summarized in Box 1, and also provide a framework for the subsequent discussions in this editorial.

**Box 1. Aspects of people centred health systems (PCHS)**

**Putting people’s voices and needs first**

PCHS are ultimately shaped by community voices and needs. Participatory governance mechanisms can channel the power of communities to mould health systems in the public interest, and hold them accountable. People-centred governance can also confront entrenched power imbalances within health systems, and address their broader social determinants.

**People centredness in service delivery**

PCHS put people’s needs first in the design and delivery of health care and services. Important principles of this approach are quality, safety, longitudinality (duration and depth of contact), closeness to communities and responsiveness to changing requirements. Capacity building in PCHS focuses, foremost, on creating capabilities to respond to people’s health care needs.

**Relationships matter: health systems as social institutions**

PCHS are social institutions, which operate through chains of relationships between different health systems actors—including administrators, health care providers, service users and researchers—each acting in their respective contexts. As such, systems thrive on mutual trust, dialogue and reciprocity, and their effectiveness correlates to the quality of these human relationships.

**Values drive people centred health systems**

In PCHS, decision making is informed by people centred values around justice, rights, respect and equality, and the principles of primary health care. Values drive people’s decisions within the health system contributing to change, and conversely, system reforms can have impacts on people’s values within the system.

This supplement is a joint production of *Health Policy and Planning* and the organizers of the Third Global Symposium on Health Systems Research, Cape Town, 2014. Its release is timed to coincide with the Symposium, that takes as its theme, the *science and practice of people-centred health systems*. We anticipate that the supplement will inform debates in the Symposium, and also that well beyond the event, it will open up the topic for continued investigation, reaffirmation and challenge in the practical as well as the academic realms of health policy and systems.

## Putting people’s voices and needs first

People centredness ultimately directs attention to the need for spaces in which people’s voices have influence in shaping the health system that seeks to serve their interests, i.e. the public interest. The World Health Report of 2008 has suggested that people centredness is a requisite ‘value’ of a primary health care (PHC) approach, required to achieve health for all (WHO 2008). Since the era of the Alma Ata declaration on PHC, participation has been a theme of health policy debates, reflecting wider development policy trends. Current discussions on participatory governance build on these past debates. Mechanisms of participatory governance range from local health committees to national level fora where people come together to inform decision making and to hold health systems accountable, as in Brazil (Cornwall and Shankland 2008). Ultimately the purpose of such mechanisms is to give people, including and most particularly, those with the greatest health needs, the power to direct resources towards those needs. Such systems place principles of equity and inclusiveness at the heart of their decision-making practices (Commission on Social Determinants of Health [CSDH] 2008).

Establishing people centred governance processes inevitably confronts the existing power balances within health systems—including the (often disproportionate) power held by clinicians, more wealthy groups and commercial interests. People centred governance also requires actions to support social empowerment, recognizing that this is not solely a function of the health sector. These actions include not only establishing, with adequate resourcing, specific decision-making mechanisms, but also changing the way health services are organized and financed, reorienting health workers and their practices and process of communication, and strengthening leadership and management within the health system (Regional Network for Equity in Health in East and Southern Africa [EQUINET] 2007).

*RIfkin* presents a systematic review of research seeking to link community participation with improved health outcomes. She finds that the majority of studies fail to establish a link for lack of a standard definition of ‘community’ and ‘participation’. Although she identifies two reviews that link community health worker programmes with improved health service delivery, concrete causal lines remain hard to establish. The author recommends a framework that views the process of community participation as a process rather than as an input into a linear, causal pathway.

Community health workers (CHWs) have been recognized as important actors in improving broader social determinants, ensuring communities’ health rights and combating social exclusion. *Nandi and Schneider* examine the roles of *mitanin* CHWs, in influencing social determinants of health in central India. They trace how these volunteer CHWs helped combat malnutrition and violence against women in the communities in which they worked, through persistent advocacy on the issues within the community, and mobilization of women to understand and claim their entitlements and seek redress.

Citing Poteete *et al.* (2010), *Abimbola et al.* propose that the governance of ‘common property resources’ such as PHC services in Nigeria is a joint enterprise of communities and governments, and hence that individuals and communities can potentially mitigate the effects of government failure in their provision. Yet, the ability of communities to co-govern effectively necessitates a balance of formal authorization from government and official independence from governmental decision-making processes. The authors, drawing on case examples from the Nigerian PHC system, finely etch these intricacies of people-centric governance.

## People centredness in service delivery

The PCHS concept encompasses as well as extends similar thinking in the domains of health care and services. People centredness in health service delivery involves putting people first in terms of how services are designed and delivered, and not merely orienting services on the basis of diseases, or for the convenience of clinicians. The World Health Organization and its regional offices have provided various interpretations of people centredness in reference to health care and services (WHO Western Pacific Regional Office [WPRO] 2007; WHO EURO 2013; WHO 2014). Quality and safety of care, longitudinality, closeness to communities and responsiveness to users’ views and changing requirements emerge as potentially important principles of people centredness in the design and delivery of health care and services. Capacity building efforts for health service providers in low- and middle-income countries (LMICs) must align with these principles, and focus on enhancing capabilities to respond to people’s emergent health care needs.

The WHO WPRO (2007) identifies five primary challenges of a people centred approach to service delivery: quality, safety, timeliness, effectiveness, efficiency and equity, and states that a people centred approach meets these challenges by ‘recognizing that before people become patients, they need to be informed and empowered in promoting and protecting their own health. There is a need to reach out to all people, to families and communities beyond the clinical setting.’ (WHO WPRO 2007). On similar lines, *Ferrer et al.* characterize people centredness in health care to be a function of ‘longitudinality’—the depth and duration of interconnectedness between a service user and provider, irrespective of illness episodes. The authors contrast this with a traditional biomedical approach in which health care programmes are designed to respond to the frequency of diseases and underlying risk factors.

Building on the importance of the interconnectedness between service users and providers, *Manu et al.* present a qualitative assessment of community-based ‘surveillance volunteers’ (CBSVs) in the setting of a cluster-randomized trial. CBSVs in the intervention zones were trained to promote essential newborn care practices through home visits to assess newborns for danger signs and refer to health facilities. This intervention reflected an approach of ‘delivery of care as close to home as is safe and cost-effective’ (WHO EURO 2013). They found that mothers of newborns found to be at risk, and who were provided with a referral card, had a greater perception of recognition of their entitlements. Yet, the unpreparedness of health centre staff to receive these mothers and their newborns meant that some were not treated with adequate respect in the facility.

*Asfaw et al.* argue that patients’ views and levels of satisfaction have rarely been taken into consideration, in the context of important health service reforms. In their study of patient experiences of task-shifting reforms in Ethiopia, they found that users of anti-retroviral therapy services treated by nurses and health officers were significantly more likely to report satisfaction than those who received services from doctors. Based on their findings, and supported by previous research in Ethiopia, they propose considering task shifting as an important mechanism towards scaling up towards Universal Health Coverage, with particular value in underserviced areas.

## Relationships matter: health systems as social institutions

People centredness is also about recognizing that health systems are social institutions, in which different health systems actors—including administrators, health care providers, service users and researchers—are linked to each other in chains of relationships, with each acting in a complex of social, organizational and economic contexts (Gilson 2003; UN Millennium Project 2005). When we see systems as social institutions primarily defined by the people who constitute them and their human relationships, the ways of bringing about change in health systems go beyond altering written rules and distributing resources, and extend to managing these chains of relationships effectively. A range of such interventions are highlighted by the papers cited in this section, including innovations to strengthen managerial practice and recruit managers, encouraging a system of accountable multi-level governance and a focus on improving gender relations within the health system.

The article by *Abimbola et al.* applies the multi-level governance framework developed by Nobel Laureate Elinor Ostrom *et al.* (1994) to the context of PHC governance in Nigeria. An emerging observation from this analysis is that optimal delivery of PHC services depends on the strength and nature of relations among all health systems actors. On a similar note, *Nandi and Schneider* report from their Indian study that the *mitanin* CHWs’ engagements and interactions with health service providers and efforts to revitalize local political structures played a key role in villagers receiving the health services to which they were entitled.

*Aberese-Ako et al.* in their Ghanaian study of frontline health worker motivation, highlight the interconnectivity of relationships between the health administration, health workers and patients. Injustice and disrespect towards health workers by the administration are widespread and have varied manifestations, and have a profound influence on the workers’ approach to their professional commitments and to patients. Echoing these findings, *Namakula et al*., reporting on conflict and post conflict experiences of health workers in northern Uganda, observe that the workers’ motivations to remain in service are frequently determined by their relationships with local communities and their co-workers. *Daire and Gilson*, meanwhile, focus on a neglected group of people in the system—PHC facility managers—and, in an urban South African setting, explore the factors influencing their leadership of people and activities. The authors describe strategies to encourage managers’ reflective capacities to support them in transitioning from a nursing identity to the leadership identity needed to manage the people and relationships that underpin all aspects of health facility management.

The link between knowledge and policy in the health system is a poorly explored one, and *Corluka et al.* begin to bridge this gap, importantly by treating researchers as an integral part of the health system, in an Argentinian study that investigates relationships between health researchers and policymakers. The authors found that a range of relational factors including reduced opportunities for interaction, cultural obstacles, differing frames and worldviews, and mistrust impeded the effective translation of knowledge into policy. *Scott et al.* take forward the theme of the researcher being an integral part of the system, reporting from the same project as *Daire and Gilson*. Their participatory method is inseparable from the intervention, in which health systems researchers and health system managers worked together to understand and address the relationship challenges underlying weak co-ordination among health system actors in the district health system.

## Values drive people centred health systems

Values are important drivers of change within the health system, and conversely, system reforms can have impacts on values within the system. *Aberese-Ako et al.*’s poignant accounts of the injustices and disrespect experienced by Ghanaian health workers from their health administration are a testament to how devaluation of health systems by upstream decision makers can influence the performance of a health service. The ‘internal’ (to providers) and ‘external’ accountability (to patients) of a health system are inseparable, suggest the authors. Social values also crucially shape identities of people within the health system. *Daire and Gilson* observed that senior nurses who had reached the positions of facility managers still saw themselves more as clinical care providers (nurses) than as managers, and this led them to neglect their strategic and leadership roles in the system. Organizational environments also often impeded their attempts to practise leadership, an observation with wide relevance for LMIC health systems seeking to build leaders from within.

Supporting change in health systems in the ways outlined in previous sections requires consideration of what values should drive decision making in a people centred health system. Respect for, and achieving equal treatment of people of different genders, religious persuasions, social groups and economic strata are important principles in considering how services should be planned and delivered. people centred service delivery, meanwhile, as highlighted by *Ferrer et al*., for instance, once more emphasises the importance of the values and principles of PHC, notably first contact care when needed, person-focused care over time (longitudinality), and comprehensiveness and co-ordination of services (Starfield 2009).

Values such as justice clearly flow through different levels of a people centred health system, and define its overall culture, and the extent to which it commands the trust of communities. As already noted, taking account of people implies the need to engage them in decision making about how to direct resources for health—in turn, highlighting the importance of procedural justice as a complement to distributional justice, in a people centred system (Mooney 2009). Recognizing relationships matter, meanwhile, directs attention to the importance of trust and trustworthiness as a basis for building those relationships and supporting co-ordination among health system agents (Gilson 2003).

Acknowledging the human values linked with people centredness ultimately may also provide a yardstick against which to assess actions and decision in health systems. For example, we may ask—how do new approaches to funding or resource allocation impact on procedural justice, trust or continuity of care, and resultantly, how do they build or undermine people centredness?

## People centred science

The articles in this supplement showcase advances in the field of health policy and systems research (HPSR), emphasizing different ways of doing research on health systems that focus on people and understanding them, that seek to support them and that challenge the researchers themselves to see their role in the system. Measures of quality in HPSR can be distinct from other forms of health research. It is particularly pertinent to address questions and themes that are relevant to people trying to bring about change in health systems in their specific contexts, and also to ask the right types of questions that support such positive change (Sheikh *et al.* 2014).

*RIfkin’s* review highlights that inappropriate conceptual frameworks and methods underlie failure of the most common type of research identified, the randomized controlled trial, in making the link between community participation and health outcomes. The author identifies as a weakness of the approach, the assumption of a linear relationship between community participation and health outcomes, and inadequate processes for identifying and collecting data on context, including the history and culture of the community and social determinants of health. The review underscores that the tendency to focus on a simplistic ‘what works?’ principle does not adequately contribute to understanding ‘how’ participatory processes can develop community ownership and resultantly contribute to health improvements. *RIfkin* also points to the importance of dialogue and participation in the research process, illustrating the value of involving community members in ‘designing, implementing and evaluating specific health interventions.’ *Scott et al.* make a significant contribution to the methodological literature on action learning in health systems (Lehmann and Gilson in review). Paraphrasing the authors—they focused on ‘learning “with” rather than “about” health systems actors in cycles of action and reflection over a prolonged period of time’, as part of their exploration of the nature of governance in district health systems. In doing so, they underscore multiple opportunities for a transformative role for health policy and systems researchers in a health system.

Health policy and systems researchers produce knowledge as part of an interactive enterprise along with other health system actors, based on dialogue, trust and shared commitment to change. Several papers in this supplement reflect such a culture of co-production of knowledge, and reinforce the importance of health policy and systems researchers as important and integral actors in, and of contextually relevant, values-driven research knowledge as a crucial currency of, people centred health systems. This supplement brings together a unique collection of research papers that use such approaches to explore people centredness across a variety of LMIC health systems, and contributes to an exciting new dynamic in the field of HPSR.
